# Development of a Falls Efficacy Scale: A Contextually Relevant and Appropriate Tool for Indian Seniors

**DOI:** 10.7759/cureus.95574

**Published:** 2025-10-28

**Authors:** Palani G Kumar, Sarvang Desai

**Affiliations:** 1 Department of Physiotherapy, Sumandeep Vidyapeeth Deemed to be University, Vadodara, IND; 2 Department of Orthopaedics, Sumandeep Vidyapeeth Deemed to be University, Vadodara, IND

**Keywords:** community-dwelling older adults, elderly falls, fall-risk assessment tool, falls efficacy scale, falls prevention, falls rehabilitation, geriatric fall, geriatric physical therapy, india, risk of fall

## Abstract

Introduction

India is expected to have one of the largest elderly populations in the world by 2050, with approximately 20% of the total population projected to be senior citizens. Currently, fall efficacy scales do not include all the relevant activities, and some daily activities performed by seniors at risk of falls are not included in them. Therefore, it is important to have a fall risk assessment screening tool that is contextually appropriate for the Indian elderly population.

Methodology

A literature review, opinions from eight multidisciplinary experts and an interview with 104 senior participants were held. Based on this, the activities performed by seniors at risk of falls were identified. Consensus among experts was sought in finalising the items, and then the scale was pilot-tested with seniors.

Results

A total of 22 items were pooled, comprising 14 items identified through interviews with seniors, three new items suggested by experts, and five items from existing scales. Some items were added with additional conditions. The item-level Content Validity Index (CVI) was between 0.86 and 1, and the full-scale CVI was 0.95. After the pilot testing, 20 items were included in the final scale.

Conclusion

The newly developed scale is expected to address the current gap of a contextually relevant falls efficacy scale in India. Future studies on test-retest reliability and content validity of the new scale are warranted.

## Introduction

The decade from 2021 to 2030 has been designated by the United Nations General Assembly (UNGA) as the Decade of Healthy Ageing [1]. In India, anyone aged 60 or above is regarded as a senior citizen [2]. Reports from the United Nations Population Fund (UNFPA) & the International Institute for Population Sciences (IIPS) estimate that India will have approximately 173 million senior citizens by 2026, rising to 198 million by 2030 and 326 million by 2050 [3].

The World Health Organization (WHO) reports that falls are among the leading causes of unintentional injury deaths worldwide. It estimates that around 80% of the 684,000 individuals who die from falls globally each year are from low- and middle-income countries, with adults aged 60 years and older accounting for the highest proportion of fatal falls. Additionally, approximately 37.3 million falls require medical attention each year [4].

A fall is defined as an event that results in a person coming to rest inadvertently on the ground or floor or other lower level [4]. Major health challenges of ageing, also known as "modern geriatric giants," comprising frailty, sarcopenia, anorexia of ageing, and cognitive impairment, can lead to falls [5,6]. Early detection and intervention of these challenges can help in reducing disability, hospitalization, and mortality. Fear of falls is considered one of the intrinsic factors that increases the risk of falls in the elderly [7]. It leads to self-avoidance of physical activities and reduces confidence in performing daily essential tasks.

Bandura defines self-efficacy as an individual’s perception of capabilities within a particular domain of activities [8]. A person with low perceived self-efficacy tends to avoid certain activities due to the fear of falling. To assess fall-related fear, Tinetti et al. developed the Falls Efficacy Scale (FES), primarily targeting frail elderly individuals by evaluating their indoor activities [9]. Hill et al. then modified it and developed the Modified Falls Efficacy Scale (MFES) by including outdoor activities [10]. To make the assessment more universal, the Falls Efficacy Scale-International (FES-I) was developed and validated among many cultures [11].

Despite these efforts, many items on these scales have remained specific to the Western population. While translating and validating the MFES into Hindi and Gujarati languages, the authors found that certain activities can not be applied to Indian seniors. In contrast, some activities that Indian seniors tend to avoid due to fear of falls are not included [12]. Also, these scales are at least two decades old and need a revisit. Furthermore, a few activities, such as answering phone calls, have become obsolete with the advent of mobile phones, even in the Western population. All the above warrant the development of a falls efficacy scale that is contextually relevant to Indian senior citizens.

## Materials and methods

The Sumandeep Vidyapeeth Institutional Ethical Committee approved this prospective and exploratory study (approval no. SVIEC/ON/PHYS/PHD/16/NOV/1717020). The study was also registered with the Clinical Trials Registry India (CTRI) under the registration number CTRI/2018/05/013809 on May 11, 2018. The scale was developed in four stages. The literature review served as the first stage, followed by interviews with seniors to identify obsolete items in the MFES and those items that give rise to fear of falls in the elderly but are not included in it. The third stage involved obtaining the opinions of multidisciplinary experts and reaching a consensus on developing a new falls efficacy scale. The fourth and final stage was pilot testing, which was done using a small and convenient sample size.

A literature review was conducted to identify scientific literature on falls efficacy scales specific to the Asian elderly. It was done using the following keywords: falls efficacy scale, fear of falling, Asian elderly, ageing and mobility, balance in geriatric care. The search was conducted in PubMed, Cumulative Index to Nursing & Allied Health Literature (CINAHL), and Physiotherapy Evidence Database (PEDro). It yielded three studies that had been conducted among the elderly in China, Taiwan, and Hong Kong [13-15]. The items that were not listed in MFES were identified.

Using convenience sampling, community-dwelling individuals aged 60 and above who could ambulate independently with or without walking aids were approached to participate in the study. Participants were recruited until the saturation point was achieved, at which point no new activities that could cause a fear of falling were reported. Elderly subjects with or without comorbidities, who could understand either Hindi, Gujarati, or English, were included. Subjects with a score of 24 or below in the Mini-Mental Status Examination (MMSE) and or a score of six or more on the 15-item Geriatric Depression Scale (GDS) were excluded. Subjects with a history of any condition that may affect the balance and gait were also excluded.

Patient information sheets were provided, and written informed consent was obtained from each subject. Subjects were asked to complete the MFES, or were interviewed one-on-one if they did not have formal education. The validated Hindi and Gujarati versions of the MFES, along with its original English version, were utilised based on the language preferences of the participants. They were asked about their perception of the risk of falls while performing these activities and also about the frequency of performing them on a four-point Likert scale. They were asked to add any other activities, not listed in MFES, that they perceive may lead to falls.

Content validity was evaluated for the draft of the scale using ratings from a multidisciplinary panel. Ten experts were approached, and eight agreed to participate in the study. The multidisciplinary team included two orthopaedic surgeons, one general physician specialising in elderly care, one nurse, and four physiotherapists. All were post-graduates or doctoral scholars in their fields and had over 12 years of professional experience. The initial draft of the scale was developed based on a literature review and interviews with seniors. The experts provided their opinions on language clarity and suggested modifications to some activities with specific conditions. Each expert independently rated each item on a four-point scale: 1=strongly disagree, 2=disagree, 3=agree, and 4=strongly agree. The item-level Content Validity Index (I-CVI) was calculated as the percentage of experts assigning a rating of three or four to an item. The scale-level CVI (S-CVI) was calculated as the average of the I-CVIs for all items. An I-CVI of 0.78 or higher (with at least six experts' agreement) and an S-CVI of 0.90 or higher were considered indicators of excellent content validity.

The new scale was forward-translated into Hindi and Gujarati languages, but not back-translated, as the scale was still in the development stage. However, the face validity of the translation was assessed with native speakers. Pilot testing for the new scale was conducted using a convenient sampling of 15 subjects per language to ensure that all items on the scale were easy to understand in all the three languages. For pilot testing, the inclusion and exclusion criteria for participants remained the same as those used for the seniors' interview. The participants were instructed to respond to the activities on the scale only if they engaged in them regularly or frequently.

## Results

The literature review yielded three articles that attempted to identify culturally relevant activities for the Asian population [13-15]. Walking on a wet or slippery surface, getting up from the floor, squatting, and reaching for things in lower cabinets were some of the activities found in those articles and were included in the draft.

A total of 104 seniors participated in the interview, and a mixed sample design was employed to reduce potential biases related to the participants' gender and geographical location. Fifty-one male participants (49%) and 53 female participants were involved in the study. Fifty-three respondents were from urban (51%) and 51 were from rural (49%) backgrounds. The participants' mean age was 71.90 (±7.90), with a maximum of 97 years. Forty-nine subjects were between 60 and 69 years old (47%), 37 were between 70 and 79 years old (36%), and 18 were aged 80 years and above (17%). Seventy-seven seniors (74%) perceived themselves as active, whereas 27 seniors (26%) reported a sedentary lifestyle.

In the seniors’ interview, it was found that three activities in MFES were not relevant to the Indian elderly. Sixty-three seniors (61%) did not perform light housekeeping at all. Forty-one seniors (39%) agreed that they did light housekeeping, with nine of them reporting that they rarely did the activity, which significantly reduced the percentage from 39% to 30%. Although 61 seniors (59%) reported performing the simple shopping activity, 12 of them said that they did it very rarely, which drastically reduced the percentage from 59% to 47%. In the case of gardening/hanging out the washing, 70 seniors (67%) reported not doing the activity, while only 34 seniors (33%) agreed that they hung out the washing. However, none of the senior participants were involved in gardening. Preparing a meal was also less frequently performed and more gender-specific, as all 51 males (100%) reported not doing this activity. In contrast, the seniors reported perceiving fear of falling while doing certain activities not listed in MFES, such as sitting on the floor, getting up from the floor, squatting, climbing up on a stool/chair for reaching/cleaning, and performing farming activities. They reported a total of 14 such activities. Three more activities were included as suggested by the expert panel, such as suddenly turning to one side while walking, picking up objects from the floor while standing, and riding a bicycle/two-wheeler.

After the omissions and additions, 28 items were pooled and reviewed by experts for language clarity, and recommended language corrections were made. Experts suggested modifications in certain activities under specific conditions, such as getting dressed and undressed in a standing position, using the bathroom without a seat or any supporting aids, and using public mass transportation. While drafting the scale, maximum attention was paid to using simple English to ensure easy understanding by the participating elders. For example, "Answer the door" was changed to "Responding to someone knocking or calling at the door," and "walking around the inside of your house" to "walking inside the house, like moving from one room to another."

After the first round, the 28 items were reduced to 22, as some items were similar in nature and content. Experts again evaluated the 22 items on a four-point Likert scale, and the CVI was calculated. Of the 22 items, the experts unanimously agreed on 14 items, while six experts approved eight items. Therefore, the I-CVI values ranged from 0.86 to 1.00. The overall CVI for the scale was 0.95, indicating excellent content validity. Details of the CVI are shown in Table 1.

**Table 1:** Item-wise description with the Content Validity Index (CVI) for the scale (with eight experts) I-CVI = Item-Level Content Validity Index; S-CVI = Scale-Level Content Validity Index (average method).

Item No.	Item description	I-CVI	Interpretation
1	Getting dressed and undressed in a standing position	0.86	Acceptable
2	Using the bathroom without a seat or any supporting aids	1	Excellent
3	Walking on wet and slippery surfaces	1	Excellent
4	Lowering yourself to the floor for reaching or sitting	1	Excellent
5	Getting up from the floor	1	Excellent
6	Get in/out of a chair	0.86	Acceptable
7	Walking inside the house, like moving from one room to another	1	Excellent
8	Climbing up or down a staircase without handrails	1	Excellent
9	Picking up objects from the ground/floor in a standing position	1	Excellent
10	Climbing up on a stool/chair for reaching/cleaning	1	Excellent
11	Responding to someone knocking/calling at the door	1	Excellent
12	Walking on an uneven surface	1	Excellent
13	Walking through an area that is cluttered or has obstacles	0.86	Acceptable
14	Walking in the area nearby	0.86	Acceptable
15	Suddenly turning to one side while walking	0.86	Acceptable
16	Using public mass transportation such as a bus	1	Excellent
17	Crossing a road/street with minimal traffic	1	Excellent
18	Riding a bicycle/two-wheeler	1	Excellent
19	Riding a bicycle/two-wheeler with a passenger in pillion	1	Excellent
20	Going out for a social event (e.g. going to a worship place or attending a family gathering)	0.86	Acceptable
21	Going out for a simple shopping or dropping off/picking up the grandchild	0.86	Acceptable
22	Carrying out the farming activities	0.86	Acceptable
	S-CVI	0.95	Excellent

A pilot test was conducted with 45 participants, 15 each for English, Hindi, and Gujarati, to evaluate the clarity and applicability of the 22 activities in the scale. Twenty-five male participants and 20 female participants attempted all the activities. Thirty of them were from rural areas, while 15 were from urban backgrounds. The frequency and percentage of participants who completed each activity are presented in Table 2.

**Table 2:** Results of pilot testing of responses to activities among seniors (N=45) Item-wise description with response frequencies and percentages.

Activity No.	Activity description	No of seniors performing the activity (%)
1	Getting dressed and undressed in a standing position	45 (100%)
2	Using the bathroom without a seat or any supporting aids	45 (100%)
3	Walking on wet and slippery surfaces	45 (100%)
4	Lowering yourself to the floor for reaching or sitting	45 (100%)
5	Getting up from the floor	45 (100%)
6	Get in/out of a chair	45 (100%)
7	Walking inside the house, like moving from one room to another	45 (100%)
8	Climbing up or down a staircase without handrails	45 (100%)
9	Picking up objects from the ground/floor in a standing position	45 (100%)
10	Climbing up on a stool/chair for reaching/cleaning	45 (100%)
11	Responding to someone knocking/calling at the door	45 (100%)
12	Walking on an uneven surface	45 (100%)
13	Walking through an area that is cluttered or has obstacles	45 (100%)
14	Walking in the area nearby	45 (100%)
15	Suddenly turning to one side while walking	45 (100%)
16	Using public mass transportation such as a bus	45 (100%)
17	Crossing a road/street with minimal traffic	45 (100%)
18	Riding a bicycle/two-wheeler	24 (53%)
19	Riding a bicycle/two-wheeler with a passenger in pillion	24 (53%)
20	Going out for a social event (e.g. going to a worship place or attending a family gathering)	45 (100%)
21	Going out for a simple shopping or dropping off/picking up the grandchild	45 (100%)
22	Carrying out the farming activities	29 (64%)

All 45 participants (100%) reported regularly performing 19 activities listed in the 22-activity scale. Of the remaining three activities, one that was specifically related to farming, was carried out by 29 participants, representing a moderate 64% response from the total respondents; however, 28 of the 30 rural participants (97%) were engaging in this activity. Since the scale was designed to be inclusive for both rural and urban populations, it was decided to keep this item in the final version of the scale. The other two activities, riding a bicycle or a two-wheeler alone or with a pillion passenger, were also not commonly performed. Twenty-four out of 45 (53%) participants engaged in these activities, primarily male participants, with 24 out of the 25 males respondents reporting a positive response. Therefore, they were removed from the final scale. As a result, the newly developed scale consisted of 20 items.

## Discussion

Globally, FES, especially FES-I, has been translated and validated, and some cultural adjustments have been made, including in specific Indian languages [16]. However, the relevance of individual items to the lifestyle of the participants has not been assessed [17]. India, the world's most populous country, is projected to become the leading geriatric nation within three decades [3]. This will also bring in challenges associated with modern geriatric giants. With limited social security and a significant gap between urban and rural health infrastructure, the country is at a pivotal crossroads [18,19]. Therefore, creating a questionnaire to identify falls, a major geriatric challenge, across different settings is essential to initiate and support prevention strategies.

Of the 14 activities in MFES, significantly fewer Indian seniors reported regularly engaging in three activities that caused them a fear of falls: light housekeeping, gardening/hanging out the wash, and simple shopping [20]. In India, household chores such as housekeeping and hanging out the wash are typically performed by young family members or a maid if the family can afford one. Similarly, gardening is not a regular activity due to the constraints of urban space and rural lifestyles. Although seniors do go outside, simple shopping is often seen as a task mainly performed by young adults in the family.

Fourteen new items related to fear of falls, as reported in the seniors' interviews, were added. Walking on wet and slippery surfaces, climbing up on a stool/chair for reaching/cleaning, walking through an area that is cluttered or has obstacles, and carrying out farming activities were a few of them. Experts reviewed the existing FES scales and recommended the inclusion of three additional items, namely picking up objects from the ground/floor in a standing position, suddenly turning to one side while walking, and riding a bicycle/two-wheeler.

Consequently, a total of 28 items were compiled and submitted for expert opinion on language clarity, as well as for their further suggestions or corrections. Experts suggested adding specific conditions to certain activities and making them more challenging, such as changing 'take a bath or shower' to 'using the bathroom without a seat or any supporting aids' and modifying 'getting dressed and undressed' to specify 'in a standing position.' It is reported that using challenging Activities of Daily Living (ADLs) instead of basic ADLs can better differentiate self-efficacy reports of the elderly and between participants in good versus poor health [21]. Simple English was used to the greatest extent possible in the new scale for easier and more precise understanding. For example, 'Answer the door' was changed to 'Responding to someone knocking or calling at the door,' and 'Walking around the inside of your house' was simplified to 'walking inside the house, like moving from one room to another'. Similarly, in India, public transportation includes auto rickshaws, which can reach the doorstep of older people, so it was changed to using public mass transportation, such as a bus.

In the original work, Tinetti et al. assessed confidence in performing an activity on a scale of 1 to 10, where one indicated "very confident" and 10 means "not confident at all." Conversely, MFES measures are from 0 to 10, with zero indicating "not confident" and 10 indicating "completely confident." To improve understanding and clarity for the elderly individuals, it was decided to retain the 0 to 10 scale on the new scale. It was also decided to include more categories, ranging from "Not at all confident" to "Fully confident," with intermediate options such as "Fairly confident," "Moderately confident," and "Quite confident." These additional categories were included to increase sensitivity and enable greater discrimination.

Although the study attempted to identify the activities that Indian seniors might avoid due to fear of falls, it has some inherent limitations. Most participants are in the 60- to 69-year-old age group; however, studies suggest that the risk of falls significantly increases after the age of 70. Apart from age, many seniors report an active lifestyle; therefore, they would likely perceive a risk of falls when undertaking challenging ADLs rather than basic ADLs. The concept of having confidence in performing activities while avoiding falls might be unfamiliar, especially among the rural population, which could influence how they reported the activities they avoided to reduce the risk of falling. The study was conducted with senior participants primarily from the western region of India; therefore, a scale developed based on their interviews for the entire country, which is diverse in languages, customs, and activity patterns, may not be entirely appropriate. The scale needs to be translated into various regional languages of India and validated in them to make it more useful for the entire nation.

## Conclusions

As one of the world’s most populous countries, India is expected to have a large number of senior citizens soon, along with the associated health problems, including falls and their sequelae. Therefore, the country needs a scale that assesses the risk of falls in older adults for earlier identification and introduction of strategies to prevent them. The scale developed here included eight new items, partially or totally excluded three items, and simplified the language for 11 items compared to the MFES. This newly developed scale has a high CVI, indicating consensus among healthcare professionals. It is expected to fill the gap and remain broadly gender- and location-neutral, allowing it to be used for almost the entire population. The scale needs to be evaluated in future for its test-retest reliability, content validity, and predictive effectiveness in identifying potential fallers among community-dwelling elderly individuals in India, thereby establishing its validity as a screening tool.
